# Optimal H1N1 vaccination strategies based on self-interest versus group interest

**DOI:** 10.1186/1471-2458-11-S1-S4

**Published:** 2011-02-25

**Authors:** Eunha Shim, Lauren Ancel Meyers, Alison P Galvani

**Affiliations:** 1 Deparment of Epidemiology, Graduate School of Public Health, University of Pittsburgh, Pittsburgh, PA 15261, USA; 2 Deparment of Epidemiology and Public Health, Yale University, New Haven, CT 06510, USA; 3 Section of Integrative Biology, University Texas at Austin, 1 University Station C0930, Austin, Texas, 78712, USA; 4 Santa Fe Institute, 1399 Hyde Park Road, Santa Fe, NM, 87501, USA

## Abstract

**Background:**

Influenza vaccination is vital for reducing H1N1 infection-mediated morbidity and mortality. To reduce transmission and achieve herd immunity during the initial 2009-2010 pandemic season, the US Centers for Disease Control and Prevention (CDC) recommended that initial priority for H1N1 vaccines be given to individuals under age 25, as these individuals are more likely to spread influenza than older adults. However, due to significant delay in vaccine delivery for the H1N1 influenza pandemic, a large fraction of population was exposed to the H1N1 virus and thereby obtained immunity prior to the wide availability of vaccines. This exposure affects the spread of the disease and needs to be considered when prioritizing vaccine distribution.

**Methods:**

To determine optimal H1N1 vaccine distributions based on individual self-interest versus population interest, we constructed a game theoretical age-structured model of influenza transmission and considered the impact of delayed vaccination.

**Results:**

Our results indicate that if individuals decide to vaccinate according to self-interest, the resulting optimal vaccination strategy would prioritize adults of age 25 to 49 followed by either preschool-age children before the pandemic peak or older adults (age 50-64) at the pandemic peak. In contrast, the vaccine allocation strategy that is optimal for the population as a whole would prioritize individuals of ages 5 to 64 to curb a growing pandemic regardless of the timing of the vaccination program.

**Conclusions:**

Our results indicate that for a delayed vaccine distribution, the priorities that are optimal at a population level do not align with those that are optimal according to individual self-interest. Moreover, the discordance between the optimal vaccine distributions based on individual self-interest and those based on population interest is even more pronounced when vaccine availability is delayed. To determine optimal vaccine allocation for pandemic influenza, public health agencies need to consider both the changes in infection risks among age groups and expected patterns of adherence.

## Background

In response to the rapid spread of a pandemic strain of H1N1 influenza A, the World Health Organization (WHO) raised the pandemic alert to its highest phase on June 11, 2009 [1]. The H1N1 pandemic was the first influenza pandemic in over 40 years. Although most H1N1 cases in individuals were mild and the case fatality rate was lower than that of previous influenza pandemics, severe cases frequently occurred in previously healthy, young adults [2].

Vaccines hold considerable promise for reducing the spread of H1N1 influenza A. However, the H1N1 vaccine was not readily available until late October, 2009 [3]. This delayed the US vaccination program until after a large proportion of the population had already been exposed to H1N1.

There is evidence that a substantial proportion of the elderly was protected by cross-immunity from prior infection, resulting in the lowest infection rate in this age group [4]. The 2009 H1N1 influenza disproportionately affected younger patients [5,6]. The median age of hospitalized H1N1 patients was 27 years, which is much lower than the median age of hospitalized seasonal-influenza cases (between 75 and 79 years) [7,8]. Yet, H1N1 was least likely to turn fatal in patients under age 17 [8]. Such differences in age-specific susceptibility and case fatality for 2009 H1N1 strain posed a challenge to public health agencies that sought to determine optimal vaccine distribution and expected public adherence.

Determining an optimal vaccination policy can be quite challenging. An individual's risk of infection depends not only on his or her decision to be vaccinated, but also on the decisions of others [9,10]. In addition, overwhelming majority of infected people are either asymptomatic or recover without medical attention. Such cases may be unaware that they have been exposed to the virus and still seek vaccination [11]. To calculate the payoff of vaccination to an individual and to the population as a whole, it is important to incorporate the cost of vaccination as well as the benefits of vaccination such as both direct and indirect protection due to herd immunity [10,12,13].

Here, we use game theory to investigate age-dependent optimal vaccine distribution against H1N1 influenza A in the US, from both individual and population perspectives. We first model the evolving age distribution of H1N1 cases as the pandemic unfolds, and examine the optimal control strategy assuming that the vaccine becomes available before, at, or after the peak of the influenza pandemic. Then, we find the expected age-specific H1N1 vaccine allocation strategy that would emerge if individuals pursue their own interest, i.e. the Nash strategy, and compare it to a strategy that is optimal to the population as a whole, known as the utilitarian strategy. The personal payoff of vaccination varies among age groups and changes over the course of an outbreak, and we recognize that individuals may not adhere to the utilitarian strategy when acting according to self-interest.

Our game theoretical analyses of the vaccination program for an influenza A (H1N1) pandemic in the United States show that the utilitarian strategy prioritizes aggressive control among individuals of age 5 and 64 regardless of the timing of vaccination. In contrast, the Nash strategy dictates vaccination of adults, ages 25-49, as the first priority group. If the vaccination program implemented before the peak of pandemic wave, then the second priority group to be vaccinated based on the Nash strategy is preschool-age children; however, if vaccination is delayed until the peak of pandemic wave, then the second priority group is older adults (ages 50 to 64).

## Methods

To model the transmission of the 2009 H1N1 influenza and vaccination, we developed the age-structured model incorporating six epidemiological compartments (i.e. susceptible, vaccinated, latent, asymptomatic and infectious, symptomatic and infectious, and recovered). Each epidemiological compartment is then subdivided into two depending on an individual’s vaccination decision. The asymptotic dynamics of this model are then used to calculate the probability for individuals to become infected based on their vaccination decision. The expected cost of infection and vaccination associated with vaccine acceptance and refusal are calculated using these infection probabilities. Since the payoff of vaccination depends on both the individual’s decision and the population's average behaviour, we formulate our model as a population game. Monte Carlo methods are employed to determine the optimal vaccination levels driven by self-interest versus the population interest.

### Mathematical model for disease transmission and vaccination

To model H1N1 influenza transmission in the United States, we divide the population into the five age groups (0-4, 5-24, 25–50, 50–64, and 65+), according to the age classes used in US CDC case reports [14]. The numbers of people in each age group were set to values estimated for the US 2008 population (Additional File) [15]. In our model, individuals in each age class *k* are subdivided based on epidemiological status. The dynamics of influenza infection, illness, and infectiousness reflect our current understanding of the natural history of influenza. Here, subscripts *U* and *V* represent an unvaccinated and vaccinated population, respectively. We assume that *S_U,k_* (*t*), *L_U,k_* (*t*), *A_U,k_* (*t*), *I_U,k_* (*t*), and *R_U,k_* (*t*) represents the respective number of unvaccinated susceptible, latent, asymptomatic and infectious, symptomatic and infectious, and recovered individuals in age groups *k* at time *t* (*k* = 1*,* 2*, . . . ,* 5). Similarly, we define *S_V,k_* (*t*), *L_V,k_* (*t*), *A_V,k_* (*t*), *I_V,k_* (*t*), and *R_V,k_* (*t*) as the respective number of vaccinated susceptible, latent, asymptomatic and infectious, symptomatic and infectious, and recovered individuals (*k* = 1*,* 2*, . . . ,* 5).

We assume that the vaccine provides partial protection, resulting in vaccinated individuals being less susceptible than unvaccinated ones. Vaccinated individuals become infected at a fraction (1-*σ_k_*) of the rate at which unvaccinated susceptible individuals become infected, where *σ_k_* is the efficacy of the vaccine against infection for individuals of age group *k* (Additional File). We consider the three vaccination scenarios where vaccines become available before, at, or after the peak of an influenza pandemic. Thus, when vaccines become available, we assume that a proportion, *ψ_k_*, of susceptible individuals in age group *k* is vaccinated. We also assume that the same proportion, *ψ_k_*, of individuals in age group *k* who have been infected asymptomatically still may get vaccinated, because they were not aware of exposure to novel influenza A (H1N1) viruses. However, vaccine doses given to those who were already exposed to H1N1 viruses are assumed to be wasted, because these individuals already gained immunity to the H1N1 strain. Recovered individuals are assumed to be fully protected against further influenza infection for the remainder of the outbreak.

Upon infection, individuals enter a latency period, 1/*δ*. Latently infected individuals proceed to become infectious, and a proportion, *p*, of infected individuals becomes symptomatic. Infectious individuals recover after an average period of 1/*γ*. The inf luenza-induced death rates are *α_U,k_* and *α_V,k_* for unvaccinated and vaccinated individuals, respectively, for people in age group *k*. Age-specific influenza-related death rates are based on estimates of excess pneumonia and on influenza deaths from the H1N1 influenza [16]. The transmission dynamics are thus described by the following differential equations:

for *k*=1,…, 5.

We used a standard-incidence form for the force of infection *λ_k_*:

where *N* is the total population size. Thus, it follows that

 where *N_k_* is the number of people of age group *k*, i.e. 

Here *ϕ_km_* is the number of contacts per day between a person in age group *k* with people in age group *m*, and *β* is the probability of infection for a susceptible person who has contact with an infectious person.

As both epidemiological and serological data are suggestive of residual immunity to H1N1 among adults and seniors, we assume that a proportion (*ξ_k_*) of individuals in age group *k* is immune to H1N1 viruses [4]. The residual immunity incorporates the fact that younger people are more susceptible to the current H1N1 strain than older people due to lack of exposure to a similar virus in the past [4,17]. The demographic effects of aging, birth, and death by causes unrelated to influenza are not included because we only model one influenza season, where these demographic effects are negligible.

The epidemic is initiated with a proportion of each age group assumed to be immune to infection, with one person of each age group assumed infectious, and with the remaining population assumed susceptible. That is,

We assume that an influenza pandemic approaches its peak at time *t*=*ω*, and a proportion of the population is vaccinated at time *τ=ω±θ* where *θ=*0 or 21 days. We assume that vaccination instantaneously protects people, so that the state variables change discontinuously at *t*=*τ*:

with the other state variables remaining the same.

**Figure 1:**
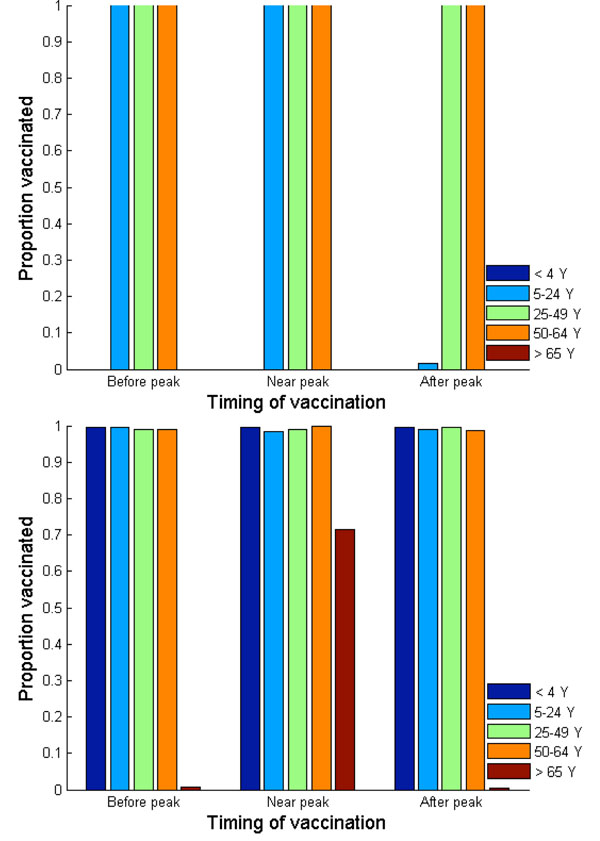
**(a) Nash and (b) utilitarian strategies when basic reproductive ratio is 1.6.** Vaccination is assumed to be offered free of charge. Vaccination is implemented three weeks before, exactly at, or three weeks after the peak of a pandemic influenza.

We further assume that the basic reproductive number (*R*_0_), defined as the number of secondary cases caused by a single infective case in a completely susceptible population, was 1.4, as estimated for the novel swine-origin H1N1 influenza outbreak [18]. For sensitivity analysis, the basic reproductive ratio was increased from 1.4 to 1.6 (Figure 1). We parameterized age-specific contact rates, *ϕ_km_*, using data from a large-scale survey of daily contacts [19]. These contact data show strong mixing between people of similar ages and moderately high mixing between children and people of their parents’ ages [20]. Given the contact data and US population size, we reconstructed the contact matrix to match our five age groups [19,20]. Using the relative size of the age group *m* (*N_m_ / N*) and the number of contacts per person in age group *k* with people in age group *m*, *c_km_*, we define the elements of the contact matrix by . To ensure that the number of contacts between age groups is symmetric, *N_m_c_km_ = N_k_c_mk_*, i.e., *ϕ_km_ = ϕ_mk_*, we made further adjustment, , and used *ϕ_km_* to be the contact matrix.

### Cost parameterization

**Figure 2:**
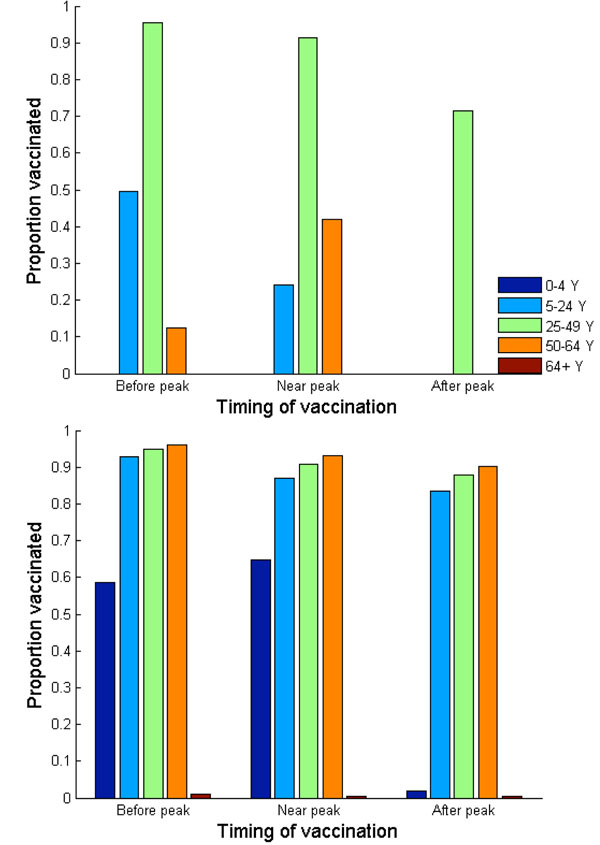
(a) Nash and (b) utilitarian strategies when vaccination administration costs $10

**Figure 3:**
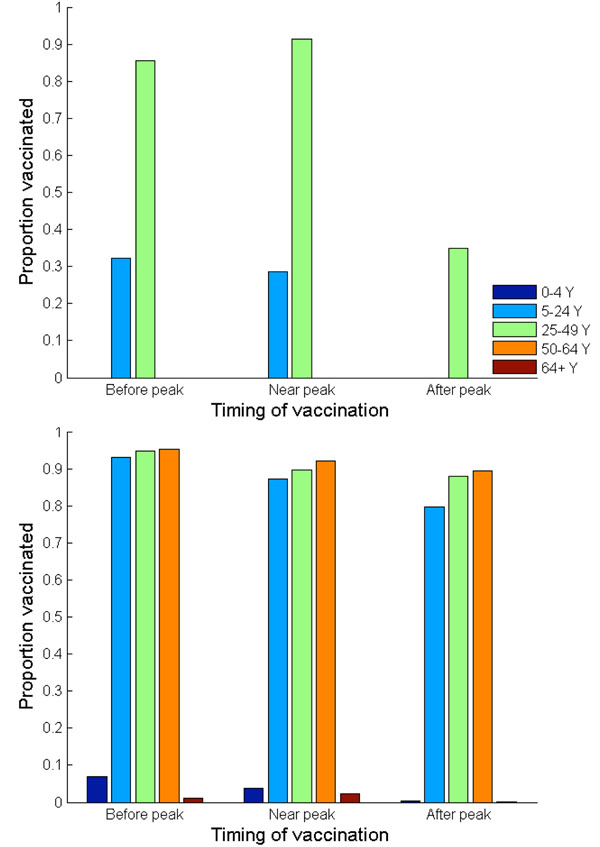
(a) Nash and (b) utilitarian strategies when vaccination administration costs $20

To calculate the average individual net payoff of vaccination strategy, we incorporated the costs associated with infection, vaccination, and the side effects of the vaccine (Table 1). We calculate the cost of infection using weighted average of the costs associated with possible infection outcomes such as mortality, hospitalization, outpatient visits, and cases without medical care. The cost of vaccination includes the value of an individual's time receiving it ($16), and travel cost ($4), resulting in the total cost of vaccination estimated at $20 [21]. The cost of administration is not included in baseline parameters because vaccine for the 2009 novel H1N1 influenza was provided free of charge in the US. However, for sensitivity analysis, we increase the cost of administration from $0 up to $20, in order to examine the elasticity of the Nash and utilitarian strategies to a range of vaccination cost (Figures 2 and 3).

**Table 1:** Parameterization of infection and vaccination cost

Variable	Base case	Reference
**Cost of vaccine side effects, $**		
**The cost associated with mild to moderate vaccine side effects per vaccinee, $**	1.37	[40] (see Methods for calculation)
**The cost associated with severe side effects (treated in ICU) per vaccinee, $**	0.78	[21,41] (see Methods for calculation)
**Costs of vaccination, $**		
**Patient time, $**	16.00	[25,42]
**Travel cost, $**	4.00	[25,42]
**Illness without medical care, $**	201(individuals under age 65); 327 (individuals over age 65)	[26,35,43,44]
**Health care costs, $**		
**General medical hospitalization**	5,861(individuals under age 65); 7,653 (individuals over age 65)	[26,35,45]
**Outpatient visits**	322(individuals under age 65); 458 (individuals over age 65)	[26,35,43,44]
**Mortality, $**	1,045,278	[26,35,45]

We calculate the cost for vaccine side effects based on the reduction in quality of life and the costs of treating individuals with severe side effects. Mild to moderate side effects are reported to occur at a probability of 5% and to reduce the quality of life by 0.05 for two days on average [21]. To calculate the cost of vaccine side effects, we use the conversion that a quality-adjusted life year (QALY) is monetarily equivalent to $100,000 [22-24]. Thus, the cost associated with mild to moderate vaccine side effects can be estimated at

0.05(0.05)(2/365)($100,000)=$1.37.

In line with clinical data, severe vaccine side effects are assumed to occur at a probability of 0.001% and result in hospitalization for 7 days, and the cost of hospitalization in ICU to treat severe side effects is taken as $3,739.05 per day [21]. In addition, we assume that severe vaccine adverse effects result in death at 5% of probability [21]. We assume that all individuals value their life equally, irrespective of their age. Thus, the value of life is estimated at $1,045,278 using average expected future lifetime earnings for all ages [25,26]. Estimating the value of life at $1,045,278, the cost of severe side effects is calculated as

10^-5^($3739(7)+0.05($1045278))=$0.78.

### **Payoff to vaccination strategy**

In our vaccination game, the payoff to an individual choosing a particular strategy depends on the average behavior of the population. We considered the two basic strategies, “vaccinator” (obtain vaccination) and “non-vaccinator” (decline vaccination). For both strategies, the payoff to an individual is measured in terms of a monetary cost due to infection and/or vaccination, based on the probability of infections and vaccine risks (Table 1 and Additional File). We also parameterized the payoff calculations with age-specific distributions of vaccine efficacy in reducing influenza morbidity and mortality (Additional File).

The net payoff to vaccinator strategy then is

where *x_k_* is the probability of infection among vaccinators, *z_M_* is the probability of mild to moderate side effects, and *z_S_* is the probability of severe side effects. *C_IV,k_* denotes the cost of infection among vaccinators in age group *k*, *C_V_* denotes the cost of vaccination, and *C_M_* and *C_S_* denote the cost of mild and severe side effects associated with vaccination, respectively.

As the vaccine efficacy is imperfect, the vaccinator may still be infected with reduced probability of infection (*x_k_*), which depends on both vaccination probability of age group *k* (*ψ_k_*) and on vaccination probability across all age groups . If infected, vaccinated individuals incur lower infection cost (*C_IV,k_*) than unvaccinated ones. The probability of symptomatic infection among vaccinators in age group *k* who are not yet infected before vaccination is given by.

Here represents the number of cumulative symptomatic infections until time *t = τ*. People who had have been symptomatically infected would be aware that they gained immunity against H1N1, thus would not get vaccinated, and therefore the expression, , represents the maximum number of vaccinating people in age group *k.*

The net payoff to a non-vaccinator is

where *C_IN,k_* denotes the cost of infection among non-vaccinators of age group *k*, and *y_k_* is the probability of symptomatic infection among non-vaccinators, given by.

Here,  describes the cumulative number of symptomatic infections among unvaccinated individuals in age group *k* after vaccination is implemented at time *t = τ*.

### Defining the Nash strategy

For individuals driven by self-interest, game-theoretic decisions are assumed to settle to a Nash equilibrium at which it is impossible for a few individuals to increase their payoffs by switching to a different strategy [27]. We define these individual decisions at the Nash equilibrium as the Nash strategy. A pure vaccinator strategy cannot be the Nash equilibrium, because when the population vaccine coverage is 100%, an individual who chooses a non-vaccinator strategy reaps the benefits of herd immunity without paying for vaccination and without experiencing possible vaccine side effects. By comparison, a non-vaccinator can result in an individual optimum under certain conditions, such as when the infection risk is sufficiently low when vaccines become available. In our age-structured model, it might be best for some people in an age group to be vaccinated and for others in the same group to choose not to get vaccinated. To allow this scenario, we consider mixed strategies whereby individuals in age group *k* choose the vaccinator strategy with probability *ψ_k_* (0 <*ψ_k_* < 1) and the non-vaccinator strategy otherwise. If all individuals play the mixed strategy *ψ_k_*, then a proportion *ψ_k_* of the population in age group *k* is vaccinated. The individual optimum can be found by solving for *ψ_k,ind_* in the equation *U_vac,k_* = *U_nonvac,k_* (*k*=1…5). The individual optimum (*ψ_k,ind_*) predicted by this game-theoretical analysis corresponds to the level of coverage *ψ_k,ind_* expected under a voluntary program where individuals act in a rational way to maximize their payoffs.

### Defining utilitarian strategy

From the perspective of group interest, the objective is to maximize the total payoff of vaccinators and non-vaccinators. If *ψ_k_* is the proportion of the population in age group *k* that is vaccinated, we can express the expected payoff  due to vaccination and an influenza pandemic as We now maximize *T*(*ψ*_1_, *ψ*_2_, *ψ*_3_, *ψ*_4_, *ψ*_5_) on the parameter space {(*ψ*_1_, *ψ*_2_, *ψ*_3_, *ψ*_4_, *ψ*_5_) | 0 ≤ *ψ_k_* ≤1} to determine the utilitarian strategy (*ψ*_1_*, *ψ*_2_*, *ψ*_3_*, *ψ*_4_*, *ψ*_5_*), which is the coverage level that would maximize the total payoff.

## Results

### Epidemiological impact of the 2009 H1N1 influenza pandemic

**Figure 4:**
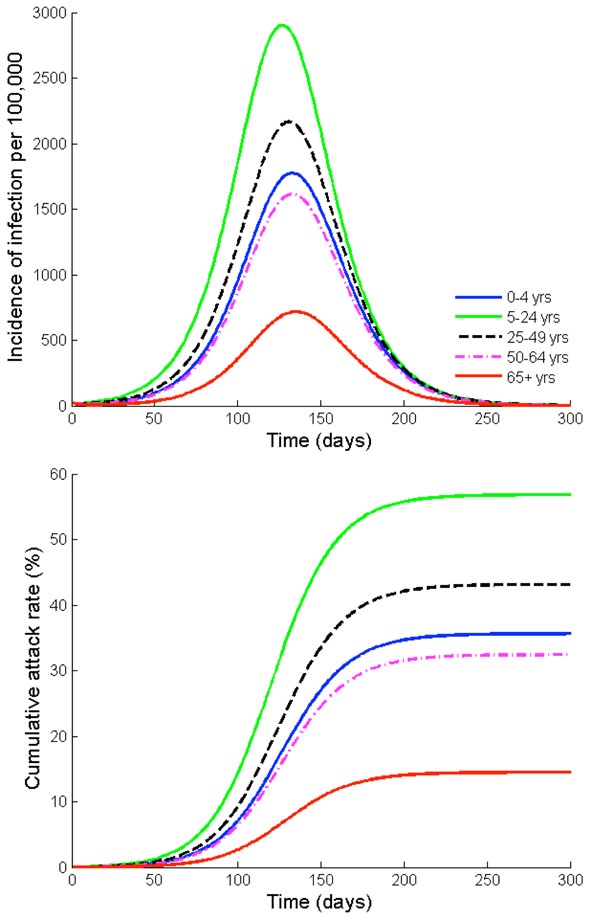
**Outcomes of influenza A/H1N1 infection.** (a) Simulated age-stratified daily influenza A/H1N1 infection incidence per 100,000 individuals in the absence of vaccination or other interventions (b) Simulated age-specific attack rates, in the absence of vaccination or other interventions. Both symptomatic and asymptomatic cases are shown.

**Figure 5:**
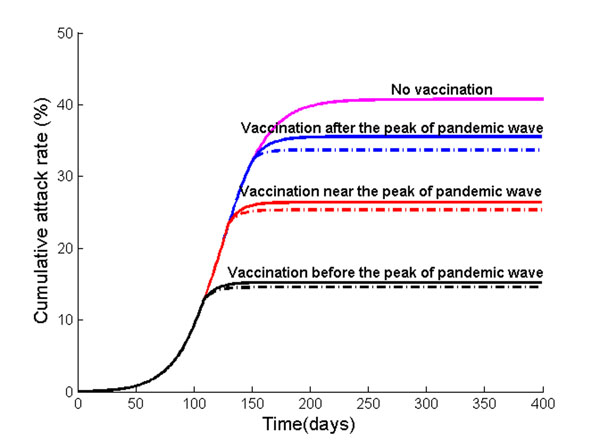
**Cumulative incidence of influenza A/H1N1 when vaccination is guided by the Nash or utilitarian strategies.** Vaccination is implemented at free of charge the three weeks before, exactly at, or three weeks after the peak of a pandemic influenza. Solid lines show the cumulative attack rate when vaccination is in alignment with utilitarian strategies when vaccination and is offered free of charge, whereas dotted lines show the cumulative attack rate assuming the vaccination is in alignment with the Nash strategies. For comparison, cumulative incidence without vaccination is also shown.

Our age-structured model of influenza transmission predicts that 41% of the US population will be infected with pandemic H1N1 influenza in the absence of interventions (Figures 4 and 5). Based on our assumptions that on average 33% of infected people become symptomatic after three day of incubation period [28], we estimate that 13% of the population will be symptomatically infected during the current influenza pandemic, which is consistent with the estimate of previous modeling studies [29-31]. However, the age-specific attack rates are predicted to vary considerably between age groups because of age-dependent activity patterns and immune profiles. The highest incidence is predicted to occur in individuals of age 5-24, followed by adult population of age 25-49, with symptomatic plus asymptomatic attack rates of 57% and 43%, respectively (Figure 4b). The lowest attack rate (14%) is predicted to occur in the oldest age group (age 65 and older).

Our results also suggest that individuals in each age group reach their highest incidence at different times (Figure 4a). That is, school-age children and young adults (age 5-24) reach their maximum incidence first, followed by adults (age 25-49) and preschool-age children under five years of age. In contrast, the oldest group is the last one to reach maximum incidence.

**Figure 6:**
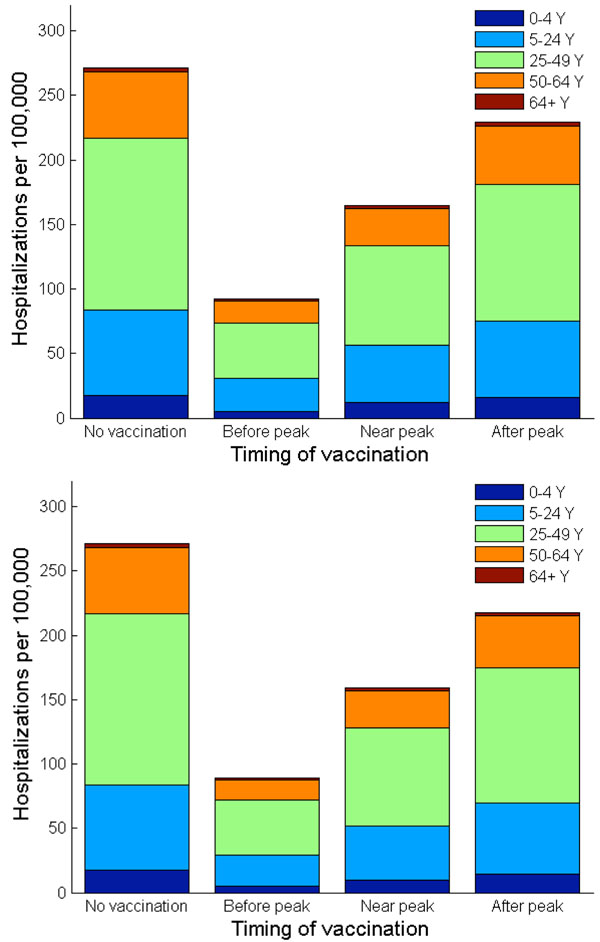
**Hospitalizations per 100,000 when vaccination follows the Nash and utilitarian strategies.** The number of hospitalizations per 100,000 is shown in age groups with and without vaccination. (a) Vaccination is implemented according to the Nash strategy at different timing of a pandemic wave, i.e. three weeks before, exactly at, or three weeks after the peak of a pandemic influenza. (b) Vaccination is implemented according to the utilitarian strategy at different timing of a pandemic wave, i.e. three weeks before, exactly at, or three weeks after the peak of a pandemic influenza.

**Figure 7:**
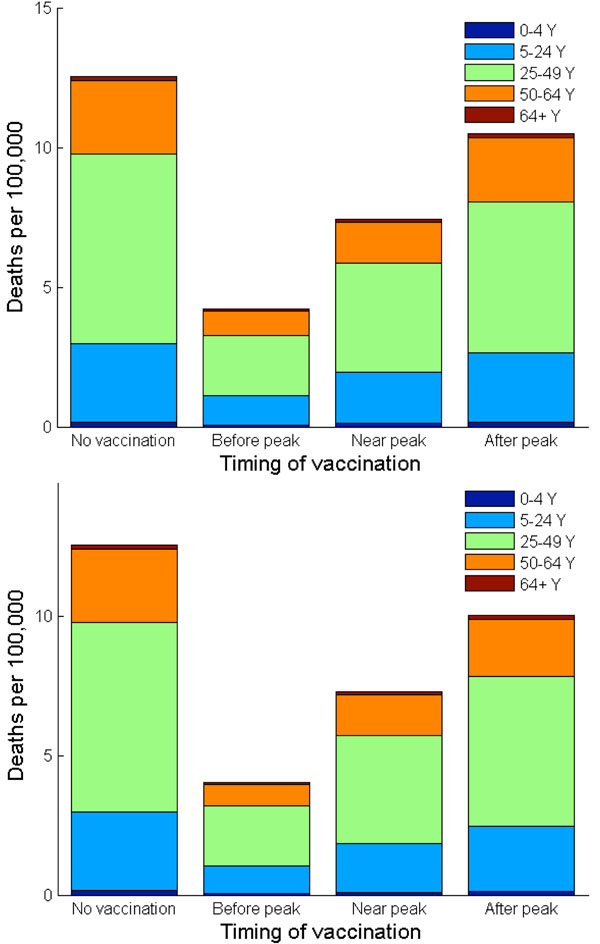
**Deaths per 100,000 when vaccination follows the Nash and utilitarian strategies.** The number of deaths per 100,000 is shown in age groups with and without vaccination. (a) Vaccination is implemented according to the Nash strategy at different timing of a pandemic wave, i.e. three weeks before, exactly at, or three weeks after the peak of a pandemic influenza. (b) Vaccination is implemented according to the utilitarian strategy at different timing of a pandemic wave, i.e. three weeks before, exactly at, or three weeks after the peak of a pandemic influenza.

In the absence of vaccination or other interventions, our model predicts that the 2009 H1N1 influenza pandemic would result in 271 hospitalizations and 13 deaths per 100,000 individuals. With their highest case fatality and hospitalization ratios among all age groups, adults (age 25-49) bear the highest case fatality rate (7 out of 13 per 100,000) and hospitalization rate (133 out of 271 per 100,000), followed by school-age children and young adults of age 5-24 (Figures 4, 6, 7).

### Optimal H1N1 vaccine distribution based on individual self-interest

**Figure 8:**
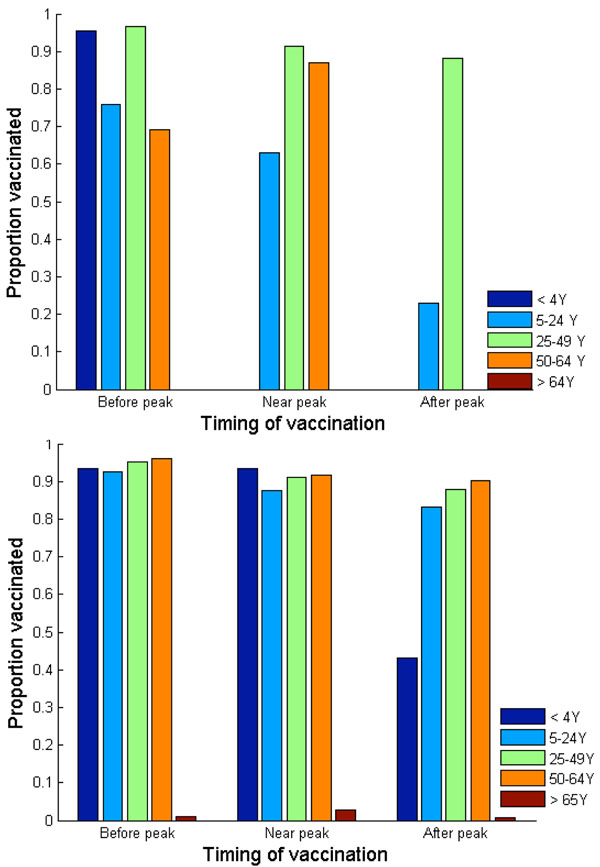
**Nash (a) and utilitarian (b) strategies when vaccination is offered free of charge.** Vaccination is implemented three weeks before, exactly at, or three weeks after the peak of a pandemic influenza.

To determine optimal H1N1 vaccine distributions based on individual self-interest versus population interest, we constructed a game theoretical age-structured model of influenza transmission assuming delayed vaccination. Our calculations show that when vaccination occurs three weeks *prior to* the peak of a pandemic wave, the Nash (individual-based) strategy prioritizes vaccinating adults (age 25- 49) and preschool-age children, followed by school-age children and young adults (age 5-24) and then older adults (age 50-64) (Figure 8a). The Nash vaccination strategy among senior population of age 65 and older would be to refuse vaccination. With such strategy, the vaccination program is predicted to reduce an overall attack rate from 41% to 15%, averting 8,514 clinical infections, 179 hospitalizations and 9 deaths per 100,000 individuals.

The Nash strategy, however, was found to be highly dependent on the timing of vaccine implementation (Figure 8a). If vaccine production is delayed, then the payoff to the vaccinators is diminished because of reduced risk of future infection. For example, if vaccination is implemented *at* the peak of a pandemic, the Nash vaccination strategy does not include the preschool-age children anymore. Instead, the Nash strategy is to vaccinate 91% of adults (age 25-49), 87% of older adults (age 50-64), and 63% of school-age children and young adults. This change in vaccination strategy occurs because preschool-age children have a relatively early pandemic peak and moderate morbidity compared to other age groups. Thus, when a vaccination is significantly delayed, the relative infection risk of this group is low.

The only age groups that are included in the Nash vaccination strategy regardless of vaccine delay are adults (age 25-49) and school-age children/young adults (age 5-24). In general, the demand for vaccine among adults (age 25-49) is the most inelastic to vaccine delay. The Nash level of vaccine coverage for school-age children and young adults falls rapidly with vaccine delay (Figure 8). For instance, when vaccination is delayed until three weeks *after* the pandemic peak, the Nash strategy is to vaccinate 88% of adults (age 25-49) and 23% of school-age children/young adults. This vaccination allocation would result in an overall attack rate of 36%, with 229 hospitalizations and 11 deaths per 100,000 individuals (Figures 5, 6, 7).

**Figure 9:**
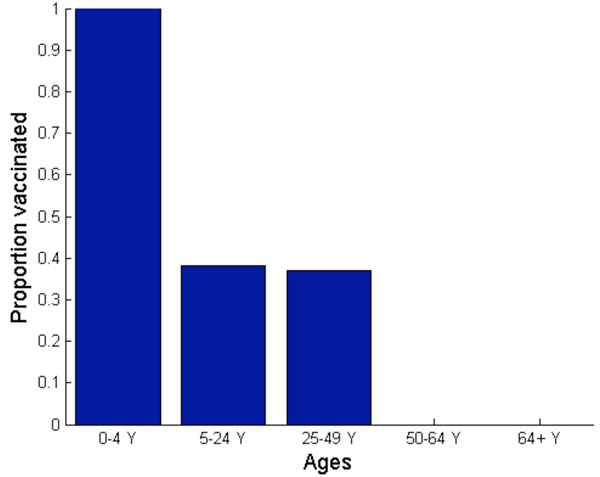
Nash strategy when vaccine is available at the beginning of an influenza pandemic and when vaccination is offered free of charge

**Figure 10:**
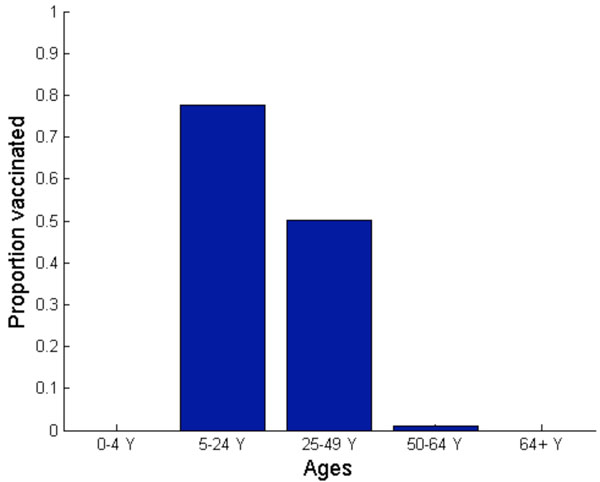
Utilitarian strategy when vaccine is available at the beginning of an influenza pandemic and when vaccination is offered free of charge

Our results also demonstrate the dependence of the Nash strategy on basic reproductive ratio of pandemic influenza. At higher transmissibility (R_0_=1.6), the Nash levels of vaccination are 100% among adults of age 25-64 regardless of timing of vaccine implementation. This is, in part, because the case fatality ratio and case hospitalization ratio are highest in these age groups, yielding a high payoff of vaccination (Figure 1). School-age children/younger adults (age 5-24), on the other hand, are expected to seek vaccination only if vaccination is offered on or before the pandemic peak (Figures 9 and 10).

Finally, we considered the rising cost of vaccination and its impact on the Nash strategy of each age group. We show that the Nash vaccination level among adults (age 25-49) is the most inelastic to the changes in vaccination cost (Figures 2 and 3). This inelasticity arises because, in this age group, the infection risk and case-fatality rate of H1N1 are high and residual immunity is low. In contrast, the Nash vaccination of older adults (age 50 to 64) is the most elastic to the changes in the vaccination cost, demonstrating the trade-off between vaccine cost and reduced benefit of vaccine due to vaccine delay. This elasticity is because the infection risk in this age group is relatively low, resulted from their residual immunity against H1N1 and low contact rate. Seniors of age 65 or older are unlikely to seek vaccination if vaccination is voluntary at a wide range of vaccination costs because their risk of infection (and thus their vaccination payoff) is lowest among all age groups.

### Optimal H1N1 vaccine distribution based on population interest

The average vaccination level across all age groups for the utilitarian strategy is higher than that for the Nash strategy (Figure 8). For example, if vaccines become available three weeks before the pandemic peak, the overall Nash and utilitarian vaccine coverage are 76% and 82%, respectively. When 93% of young individuals (age under 24), 96% of adults (age 25-64) and 1% of seniors (age 65 and older) are vaccinated according to the utilitarian strategy three weeks prior to the peak of the influenza pandemic, 25,767 clinical infections, 182 hospitalizations and 9 deaths would be averted per 100,000 individuals (Figures 5, 6, 7).

The utilitarian strategy is, however, much less effective if vaccination is delayed. For instance, if vaccination is delayed until the peak of influenza pandemic, it is estimated that 15,683 infections, 112 hospitalizations and 6 deaths would be averted per 100,000 individuals, which is considerably fewer than when the vaccination is implemented before the pandemic peak (Figures 5 and 8).

We find that the utilitarian vaccine coverage levels are more inelastic than those under the Nash strategy. For instance, if vaccination is delayed until three weeks after the pandemic peak, the resulting vaccine coverage level according to the Nash strategy falls to 37% whereas the utilitarian strategy is to still vaccinate 73% of population. Thus, the resulting disease incidence and the number of disease-related deaths are lower under the utilitarian strategy than under the Nash strategy. The utilitarian strategy includes vaccinating 90% of older adults (age 50-64), 88% of adults (age 25-49), 83% of school-age children/young adults (age 5-24) and 43% of preschool-age children. At a higher basic reproductive ratio of 1.6, the utilitarian strategy also includes the vaccination of preschool-age children (age 0-4), because the risk of infection increases with transmissibility of influenza virus, increasing the payoff of vaccination (Figure 1). However, the Nash vaccination strategy does not include preschool-age children or older adults, and thus the utilitarian coverage levels may be unachievable under voluntary vaccination.

## Conclusions

For pandemic H1N1, we find that the individual-based (Nash) vaccination strategies differ significantly from the utilitarian vaccination strategies. Without vaccination delay, the primary priority group under the utilitarian strategy is school-age children and young adults (age 5-24) because of their important role in transmitting disease (Figures 9 and 10). The case hospitalization ratio and the case fatality ratio are the highest, and thus vaccinating these individuals yields high individual and population payoffs. Indeed, regardless of length of the delay and when vaccination is guided by the Nash or utilitarian strategies, younger adults (age 25-49) are among the highest priority groups for vaccination.

However, the second priority group changes dramatically under the Nash strategy. If vaccination occurs before the pandemic peak, the second Nash priority group is preschool-age children. If vaccination is delayed, the second Nash priority group is shifted to older adults (age 50-64) or to school-age children/younger adults (age 5-24). The peak incidence among preschool-age children is relatively early compared to other age groups, thus lowering the benefit of vaccination to these children with time. Because the case fatality ratio is the highest among older adults, and H1N1 morbidity is the highest among school-age children/younger adults, the benefit of vaccination is relatively inelastic over the course of a pandemic. Therefore, the demand for vaccines among these age groups is high even if vaccination is delayed in a pandemic.

The discordance between the Nash and utilitarian strategies is even more pronounced when vaccine availability is delayed. If vaccination is delayed but implemented near the pandemic peak, the utilitarian vaccination strategy includes individuals of age up to 64, in contrast to the Nash strategy which excludes preschool-age children and older adults (age 50-64) (Figure 8). If vaccination is further delayed, the Nash strategy would also exclude adults (age 25-49), preschool-age children and older adults (age 50-64), whereas the utilitarian vaccination strategy still includes individuals of age up to 64. Therefore, the average vaccination level across all age groups at the utilitarian strategy was found to be higher than that at the Nash strategy.

Overall, our results indicate that a vaccination levels under a voluntary immunization program may not be optimal for the population, regardless of vaccine delay. Such discordance between the Nash and utilitarian strategies is predicted to be robust to the increase in the basic reproductive ratio for pandemic influenza (Figure 1). This finding is consistent with those of previous studies, which demonstrated that, in the context of vaccination against smallpox and seasonal influenza, the vaccination levels driven by self-interest are likely to be lower than those that are optimal from the population perspective [32-35].

There are three primary reasons for the discrepancy between the individual-based and utilitarian age-specific vaccination levels for pandemic H1N1. First, different age groups have different incentives to vaccinate. In particular, an earlier pandemic peak among young individuals results in a relatively low infection risk later in the pandemic compared to that for older adults. Therefore, the young are predicted to under-vaccinate under the Nash strategy relative to the utilitarian strategy when vaccination is delayed. Second, the positive externalities of indirect protection by herd immunity also contribute to the differences between utilitarian and Nash vaccination strategies. The benefits of herd immunity contribute to the utilitarian strategy, but also create an incentive for individuals to free ride on the vaccination of others. Consequently, the overall level of population vaccination is lower for the Nash strategy than for the utilitarian strategy. Third, because vaccine delivery was delayed for the H1N1 pandemic, our model predicts that people will be less inclined to vaccinate than if vaccine was available at the beginning of the pandemic. As a consequence, achieving vaccination rates high enough to achieve the utilitarian strategy may be difficult, and the discordance between the Nash and utilitarian strategies is found to increase with vaccine delay.

The guidelines for vaccinating against the 2009-2010 pandemic H1N1 influenza proposed by the CDC’s Advisory Committee on Immunization Practices (ACIP) prioritize young people aged 6 months to 25 years, who are the most efficient at transmitting influenza viruses [36]. This guideline also reflects the reduced susceptibility among the elderly due to their residual immunity from past exposure [37]. If large stockpiles of vaccines had been available prior to the pandemic, the optimal vaccine distribution strategy would be to vaccinate children in order to reduce transmission and achieve herd immunity [38,39]. However, our analysis suggests that the success of such vaccination strategies depends heavily on the timing of a vaccine’s availability. Nevertheless, our analysis might be limited by the difficulties of knowing the state of the pandemic at the time vaccines become available. In addition, our outcome measure (i.e. cost of infection and vaccination) may oversimplify the vaccination decisions or be incongruous with the consideration of the Advisory Committee on Immunization Practices (ACIP).

We found that, for both the Nash and utilitarian strategies, the optimal vaccination strategies with vaccine delay should prioritize individuals of age 25 to 49. Our results also suggest that a utilitarian vaccine strategy should also include individuals from a wide range of ages, from 5 months to 65 years; and for longer delay in vaccination, vaccination priority should increasingly be given to older individuals. Our results further suggest that age-specific demands for vaccination depend on the risk of infection at the time of vaccine delivery and the severity of the disease. When vaccination is delayed, voluntary adherence to vaccine recommendation might become lower among young individuals. This suggests that influenza pandemic response plans should include efforts to encourage the vaccination of young individuals if vaccine delivery is delayed.

## List of abbreviations

H1N1:(H: Hemaglutinin) (N: Neuraminadase); WHO: the World Health Organization; CDC: Centers for Disease Control and Prevention

## Competing interests

The authors declare that they have no competing interests.

## Authors' contributions

ES developed and analyzed the model, and carried out numerical simulations. ES produced all figures, interpreted results, and wrote the manuscript. AG suggested some of the simulations and helped write the manuscript. LM edited the manuscript. All authors read and approved the final manuscript.
